# Cardiorespiratory, enzymatic and hormonal responses during and after walking while fasting

**DOI:** 10.1371/journal.pone.0193702

**Published:** 2018-03-01

**Authors:** José Vilaça-Alves, Fernanda Muller, Claudio Rosa, Rita Payan-Carreira, Rafael Lund, Filipe Matos, Nuno Garrido, Francisco José Saavedra, Victor Machado Reis

**Affiliations:** 1 Sport Sciences Departament, University of Trás-os-Montes e Alto Douro, Vila Real, Portugal; 2 Research Center in Sports Sciences, Health Sciences and Human Development, CIDESD, Portugal; 3 Animal and Veterinary Research Center, CECAV, University of Trás-os-Montes e Alto Douro, Vila Real, Portugal; Nanyang Technological University, SINGAPORE

## Abstract

The aim of the present study was to observe whether performing a low intensity endurance exercise following an overnight fasted (FAST) or fed (FED) condition promotes different cardiorespiratory, enzymatic and hormonal responses. Nine male physical active subjects, (age 21.89 ± 2.52 years old, height 175.89 ± 5.16 cm, weight 72.10 ± 4.31 kg, estimated body fat 7.25 ± 2.11%), randomly performed two sessions of 45 minutes’ low intensity exercise (individual ventilator threshold) interspersed by seven days, differentiated only in whether they were provided with a standardized meal or not. The oxygen consumption (VO_2_) and heart rate (HR) were measured continuously at the 30-min rest, the 45-min during and the 30-min post-exercise. The testosterone (T) and cortisol (C) hormones were measured at rest, immediately post-exercise and 15-min post-exercise. The Glucose (GLU), Free fatty acids (FFA) and enzyme lipase activity (ELP) were measured at rest, 15-min and 30-min exercise, immediately, 15-min and 30-min post-exercise. Significantly lower values were observed in FED compared to FAST with: C (nmol/L) from pre (428.87 ± 120.41; 454.62 ± 148.33, respectively) to immediately post-exercise (285.10 ± 85.86; 465.66 ± 137.70, respectively) and 15-min post-exercise (248.00 ± 87.88; 454.31 ± 112.72, respectively) (p<0.05); and GLU at all times, with an exception at 15-min post-exercise. The testosterone/cortisol ratio (T/C) was significantly higher in the FED compared with FAST from pre (0.05 ± 0.02, 0.05 ± 0.01, respectively) to 15-min post-exercise (0.08 ± 0.03, 0.05 ± 0.02, respectively). No other significant differences were observed between conditions. We conclude that fasting prior to low intensity endurance exercise does not seem be advantageous, when it comes to fat loss, compared with the same exercise performed after a meal.

## Introduction

The combination of diet and physical activity is recognized as a good strategy in preventing and fighting the increasing overweight and obesity rates in the world’s population [1–4]. Recently some bodybuilding competitors and fitness enthusiasts, based on anecdotal knowledge, have been using a fat loss approach which consists of performing low intensity aerobic exercise (e.g. walking), for 20 to 60 min, after an overnight fast [5,6]. This strategy is based under the premises that: i) low or moderate intensity exercise uses predominantly fat in relation to the carbohydrates as energy substrate [7,8]; and ii) the fasting condition increases fat oxidation rates [7,8]. It would seem logical therefore, to combine both exercise and fasting in order to promote better fat loss.

However, performing aerobic exercise in fasting condition can induce fatigue and stimulation of the hypothalamic-pituitary-adrenal axis because of the low blood sugar levels [9,10]. This stimulation can increase the secretion of the steroid hormone cortisol (C) concentration [9,10]. The blood levels of C usually have a peak early in the morning and these levels are increased by the fasted condition [9]. High values of C can result in a decrease in muscle protein synthesis and increases their breakdown [10], which can promote muscle mass loss and negatively affect the fat loss process.

It is necessary to understand the endogenous hormonal response to this strategy due to their influence in the recovery phase after exercise, by modulating anabolic and catabolic processes. Cortisol and Testosterone (T) are playing a significant role in metabolism of protein as well as carbohydrate metabolism [10,11]. In fact, T could play an important role to the metabolism during the recovery phase so far, as its anabolic effect does not only concern the protein synthesis, but also seems to increased the ability of the muscle to refill its glycogen storage after exercise, through an increased activity of the muscle glycogen syntheses [11].

Furthermore, testosterone is important as a stress biomarker. Due to the anabolic nature of T and the catabolic effects of C, it’s been suggested that the ratio between the two may reflect the body’s overall anabolic–catabolic balance. An increase in T, a decrease in C, or both, could indicate a potential status of anabolism, or in the other hand, a decrease in T, a increase in C, or both, could indicate a potential status of catabolism [12].

To our knowledge only two studies were published on the effects of aerobic low-intensity exercise performed in the morning, following an overnight fast or fed condition [13,14]. However, those studies focused only on metabolic (glucose, free fatty acids) and hormonal (insulin, growth hormone and cortisol) indicators, with no measurement of the cardiorespiratory response to exercise. The aim of the present study was to observe if performing a morning low-intensity endurance exercise under fasting or feeding conditions promotes differences in cardiorespiratory, enzymatic and hormonal responses. It was hypothesized that there were significant differences between the two exercises conditions.

## Methods

### Participants

The volunteers were nine Caucasian men, age 21.89 ± 2.52 years old, height 175.89 ± 5.16 cm, weight 72.10 ± 4.31 kg, estimated body fat 7.25 ± 2.11% that practiced the exercise in the treadmill at the first ventilator threshold velocity. The inclusion criteria in the present study were regular physical activity for at least 6 months. Participants which suffered from any metabolic, endocrine or osteo-articular conditions were excluded. The volunteers were recruited among the students of a master course class. Study procedures were agreed to by all participants who provided a written informed consent to participate in the study. The study complied with the Helsinki declaration and was approved by the Institutional Review Board of the University of Trás-os-Montes and Alto Douro, Vila Real, Portugal.

### Procedures

This was a cross-sectional study. All participants filled the Par-Q test and a medical history [15]. After confirming if the participants met the inclusion criteria, they were informed not to consume alcohol or caffeine throughout all the experimental protocol and to not perform physical exercise up to the previous 72 hrs of each testing. On another day anthropometrics and skinfolds were measured, the first ventilatory threshold was evaluated and 72 hrs after, a re-test was performed. The first ventilatory threshold was measured using a ramp test in a treadmill using a portable system for pulmonary gas-exchange analysis (K4b^2^, COSMED^®^, Rome, Italy). In the ramp test the speed was 6 km.h^-1^ and inclination was increased 1% every minute until the participants were unable to walk without hand support. The lowest value of ventilation (VE) to oxygen consumption (VO_2_) ratio (VE/VO_2_) was used to mathematically calculate the velocity corresponding to the first ventilatory threshold.

The following two sessions were separated by a week and differed only in either fasting (FAST) or feeding (FED) conditions. The participants were led by a researcher to the laboratory and testing was started at 8.00 a.m. after 12 hrs of fasting. Resting metabolic rate (RMR) was measured through indirect calorimetric using the K4b^2^ device at a 30° head-of-bed elevation. Afterwards, the first blood sample was collected and the first condition of exercise was conducted (FAST or FED).

In the FAST exercise condition, participants performed 45 min of treadmill walking at a velocity corresponding to their individual first measured ventilatory threshold. In the FED exercise condition, participants consumed a standardized breakfast composed of a shake with 32g of carbohydrates (dextrose monohydrate), 24g of whey protein and 4g of fat (coconut oil), mixed with 35cl of water–subjects rested 30-min before starting the 45-min of walking. Breakfast intake was controlled by a technician.

During the walking period, blood samples were collected at 15-min and 30-min and were also collected at 0-min, 15-min and 30-min post-exercise.

In both exercise conditions, the participants remained in the laboratory for 30-min in a bed with 30° head elevation to collect oxygen consumption (VO_2_) on post-exercise recovery. During exercise and post-exercise, the VO_2_ was measured continuously breath-by-breath and then average in 20-seconds intervals. Breathing artifacts (coughing, swallowing, etc.) were detected and automatically removed by the Cosmed software when values exceeded three standard deviations from the mean. All participants reported food intake of the previous day, before each exercise condition. Participants used a wearable device to measure cardiorespiratory responses (K4b^2^). This equipment was calibrated before each test according to the manufacturer´s indications (room-air, flowmeter, delay and gas-calibrations). The ambient temperature and humidity were between 20–25°C and 40–65%, respectively, for all testing. The following were measured: VO_2_, carbon dioxide production, heart rate (HR), respiratory-exchange ratio (RER) at rest, during the 45-min walking and during 30-min after; testosterone (T) and cortisol during rest, immediately post and 15-min after exercise; glucose (GLU), free fatty acids (FFA) and the enzyme lipase (ELP) were measured at rest, 15-min and 30-min during walking, immediately post exercise, 15-min and 30-min afterwards. Substrate utilization (fat or carbohydrates) was calculated during the 45-min of walking using the formulas proposed by Frayn [16].

### Blood

After 30-min of RMR measurement, a 21/22G blood butterfly catheter (Vygon®) was inserted through on the medial cubital vein. Serum (T and C) samples were collected in 4.7 ml serum-gel tubes (S-Monovette®, Sarstedt, Nümbrecht, Germany) for the hormone measurements and for glucose (GLU). Free fatty acids (FFA) and enzyme lipase activity (ELP) were collected in 2.7 ml serum gel tubs (S-Monovette®, Sarstedt, Nümbrecht, Germany). Samples were centrifuged within 10 min from collection, at 3000 x g for 15 min; serum was harvested and stored at -20°C until assayed by a certified laboratory.

### Statistical analysis

The sample dimension analysis was performed using G*Power 3.1 software [17]. Under a framework assuming an estimation error of α = 0.05, power = 85%, having 6 measures (moments) x 2 exercise conditions, an n of 8 was necessary to reach statistical power of 95.2%. Ten subjects were initially assigned to each exercise. After drop-out and discarding of poor data, 9 valid cases were revealed.

The results are shown as means ± standard deviation and confidence interval 95% (CI95%). To assess the reproducibility of the load used for walking between the test and retest the intraclass correlation coefficient (ICC) was used. A t-test for independent measures was applied for comparison purposes between the FAST and FED conditions of exercise in the variables VO_2_ absolute and relative, HR, fat and carbohydrates used during the 45-min walking was used a t-test for independent measures. A ANOVA for repeated measures with the model: 3 times (rest, immediately post-exercise and 15 min post-exercise) x 2 conditions (FAST and FED) was used in the variables T and C; 6 times (rest, 15 min and 30 min during walking, immediately post-exercise and 15 and 30 min post-exercise) x 2 conditions (FAST and FED); and a 7 times (rest, immediately post-exercise and 5, 10, 15, 20, 25 and 30 min post-exercise) x 2 conditions (FAST and FED). A Tukey post hoc test was used to identify differences between times and sessions. All data undergoing ANOVA were tested for assumptions of normality, homogeneity and sphericity. Neither assumption was violated. The effect sizes (ES) were calculated using partial eta squared (η_p_^2^) and Cohen´s d (d = difference between means/pooled SD) for pairwise comparisons. The small, medium, and large ES would be reflected for η_p_^2^ in values greater than 0.0099, 0.0588, and 0.1379, respectively, and for Cohen´s d in values grater than 0.2, 0.5, and 0.8 [18]. The level of significance was established at 5%. Statistical analysis was conducted using SPSS 22.0 (SPSS, Inc., Chicago, IL, USA).

## Results

The ICC of test and retest used to assess the load used for walking was ICC = 1. No significant differences were observed between conditions of exercise in the variables VO_2_ absolute and relative, HR and RER (see Table 1).

**Table 1:** Mean ± standard deviation (confidence interval 95%) of the absolute (VO_2_A) and relative oxygen consumption (VO_2_R), heart rate (HR) and respiratory-exchange ratio (RER) during walking.

		VO_2_A (L/min)	VO_2_R (ml/Kg/min)	HR (bat/min)	RER
**FAST**	0-15min	1.41 ± 0.25(1.22–1.61)	19.49 ± 2.69(17.37–21.59)	99.41 ± 18.39(88.08–110.74)	0.89 ± 0.06(0.85–0.92)
	15-30min	1.43 ± 0.27(1.22–1.64)	19.74 ± 2.79(17.48–21.99)	103.93 ± 16.27(91.18–116.68)	0.92 ± 0.06(0.88–0.95)
	30-45min	1.46 ± 0.30(1.24–1.68)	20.05 ± 3.00(17.67–22.44)	106.44 ± 15.51(92.90–119.99)	0.92 ± 0.07(0.88–0.95)
	Total	1.44 ± 0.27(1.23–1.65)	19.67 ± 2.27(17.43–21.92)	103.09 ± 16.15(91.37–114.82)	0.90 ± 0.06 (0.87–0.94)
**FED**	0-15min	1.49 ± 0.28(1.30–1.68)	20.62 ± 3.23(18.52–22.72)	106.18 ± 13.26(94.85–117.51)	0.94 ± 0.04(0.90–0.97)
	15-30min	1.51 ± 0.31(1.31–1.72)	20.93 ± 3.55(18.67–23.19)	118.24 ± 19.67(105.49–131.00)	0.94 ± 0.04(0.91–0.98)
	30-45min	1.52 ± 0.33(1.30–1.74)	20.94 ± 3.71(18.55–23.32)	121.29 ± 22.23(107.75–134.84)	0.92 ± 0.04(0.88–0.94)
	Total	1.51 ± 0.31(1.30–1.72)	20.74 ± 3.57(18.50–22.99)	116.03 ± 17.01(104.31–127.75)	0.93 ± 0.04 (0.89–0.96)

FAST–Exercise performed in fasted condition; FED–Exercise performed after a breakfast.

When it comes to the oxygen consumption at the end of exercise (VO_2_ rec) no exercise effect nor an interaction time x condition of exercise was detected. However, a time effect (F_(6,96)_ = 67.239; p<0.001; η_p_^2^ = 0.808) was observed. In FAST, only the 0–5 min post-exercise presented a significantly (p<0.03, CI95% = 0.27–6.92, d = 2.53) higher value compared with resting value. In FED, VO_2_ was elevated up to 20-min after exercise (p<0.05, CI95% = 1.52–6.11, d = 1.83–4.47), compared with rest. The values of VO_2_ rec at the time 0–5 min post-exercise in both conditions were significantly (p<0.05, FAST CI95% = 0.27–6.92, d = 2.14–2.55; FED CI95% = 2.50–6.11, d = 2.50–4.47) higher compared with every other times (see Table 2).

**Table 2:** Mean ± standard deviation (confidence interval 95%) of the oxygen consumption in rest and during the 30 min of recovery (VO_2_ rec).

VO_2_ rec (ml/Kg/min)	FAST	FED
Rest	4.03 ± 0.55(3.71–4.35)	3.83 ± 0.34(3.51–4.16)
0–5 min post-exercise	7.62 ± 1.93(6.46–8.79)& **	8.14 ± 1.32(6.97–9.31)$ **
5–10 min post-exercise	4.48 ± 0.75(3.88–5.08)	5.28 ± 0.94(4.68–5.89) #
10–15 min post-exercise	4.27 ± 0.75(3.62–4.91)	5.09 ± 0.91(4.46–5.74) €
15–20 min post-exercise	3.99 ± 0.55(3.49–4.49)	5.11 ± 0.83(4.61–5.61) *
20–25 min post-exercise	4.31 ± 0.64(3.75–4.88)	4.90 ± 0.94(4.33–5.47)
25–30 min post-exercise	4.10 ± 0.70(3.50–4.70)	4.75±0.98(4.14–5.35)

FAST–Exercise performed in fasted condition; FED–Exercise was performed after a breakfast.

** p<0.05 between the time 0–5 min and the other times

& p = 0.03

$ p>0.001

# p = 0.02

€ p = 0.03

* p = 0.03 in relation to rest value

No significant differences were observed between sessions in substrate used (fat or carbohydrates) during exercise (see Table 3).

**Table 3:** Mean ± standard deviation (confidence interval 95%) of subtract used during exercise in FAST and FED conditions.

	Carbohydrates (g/min)	Fat (g/min)
FAST	1.34 ± 0.70(0.94–1.74)	0.23 ± 0.12(0.15–0.30)
FED	1.35 ± 0.38(0.95–1.75)	0.19 ± 0.09(0.12–0.27)

In relation to the hormonal responses a moment effect (F_(2,32)_ = 39.954; p<0.001; η_p_^2^ = 0.833 and F_(2,32)_ = 15.634; p<0.001; η_p_^2^ = 0.661) was observed and an interaction time x conditions (F_(2,32)_ = 13.010; p<0.001; η_p_^2^ = 0.448 and F_(2,32)_ = 7.762; p = 0.00; η_p_^2^ = 0.327) and exercise effect (F_(1,16)_ = 6.436; p = 0.02; η_p_^2^ = 0.287 and F_(1,16)_ = 8.978; p = 0.01; η_p_^2^ = 0.199), C and T/C, respectively. Immediately post-exercise and 15-min post-exercise significantly higher values of C (p = 0.00, CI95% = 65.89–295.23, d = 1.57; p = 0.00, CI95% = 105.31–307.31 d = 2.04, post and 15-min post-exercise, respectively) were found in the FAST compared with FED. The opposite was found in T/C 15-min post-exercise, with significant higher in FED compared with FAST (p = 0.01, CI95% = -0.57–-0.01, d = 1.18).

In FED, C values significant decreased from rest to the immediately post-exercise (p = 0.00, CI95% = 96.69–242.36, d = 1.40) and to 15- min post-exercise (p = 0.00, CI95% = 142.87–270.37, d = 1.69). Also, in FED a significant increase (p = 0.00, CI95% = -0.03–-0.01, d = 0.33) was observed in T/C from immediately post-exercise to 15-min post (see Table 4).

**Table 4:** Mean ± standard deviation (confidence interval 95%) of testosterone (T), cortisol (C) and ratio testosterone/cortisol (T/C) values at rest, immediately and 15 min post-exercise performed the walking exercise in FAST and FED conditions.

		Rest	Immediately post-exercise	15 min post-exercise
T(nmol/l)	FAST	19.44 ± 4.68(15.12–23.76)	22.16 ± 8.23(17.35–26.98)	22.17 ± 6.26(18.37–25.96)
	FED	20.78 ± 7.28(16.46–25.11)	17.86 ± 5.00(13.05–22.67)	19.07 ± 4.30(15.27–22.87)
C (nmol/l)	FAST	428.87 ± 120.41(333.53–524.22)	465.66 ± 137.70(384.57–546.74)	454.31 ± 112.72(382.90–525.73)
	FED	454.62 ± 148.33(359.28–549.97)	285.10 ± 85.86(204.01–366.18)*¥	248.00 ± 87.88(176.58–319.42)*$**
T/C	FAST	0.05 ± 0.01(0.03–0.06)	0.05 ± 0.01(0.03–0.07)	0.05 ± 0.02(0.03–0.07)
	FED	0.05 ± 0.02(0.04–0.06)	0.07 ± 0.03(0.05–0.08)	0.08 ± 0.03(0.07–0.10)*€**

FAST–Exercise performed in fasted condition; FED–Exercise was performed after a breakfast.

¥ p<0.00

$ p = 0.00

€ p = 0.01 between sessions

* p<0.05 in relation to rest values

** p<0.05 in relation to the time immediately post-exercise

In relation to the GLU, it was observed a time effect (F_(5,80)_ = 10.581; p<0.001; η_p_^2^ = 0.398), an interaction of time x exercise (F_(5,80)_ = 10.232; p<0.001; η_p_^2^ = 0.390) and also an exercise effect (F_(1,16)_ = 19.312; p<0.001; η_p_^2^ = 0.540). Significant, (p<0.05, CI95% = 0.90–36.75, d = 0.56–2.64), lower values of GLU were found in FED, in all times, in relation to FAST, with an exception at rest and at 15 min post-exercise. In FED the GLU values were significantly, (p<0.05, CI95% = -34.62–-6.18, d = 1.51–2.96), lower at 15 min exercise in comparison to every other times (see Table 5).

**Table 5:** Mean ± standard deviation of glucose (GLU), lipase enzyme (ELP) and fat free acids (FFA) at rest, during walking exercise and 30 min post-exercise in FAST and FED condition.

	GLU (mg/dl)	
	FAST	FED
Rest	95.00 ± 6.78(91.05–98.95)	92.11 ± 4.04(88.17–96.06)
15 min exercise	93.78 ± 8.79(86.65–100.91)	67.11 ± 11.25(59.98–74.24)*Ψ
30 min exercise	90.56 ± 5.03(86.02–95.10)	81.56 ± 7.57 (77.02–86.10)Ψ
Immediately post	92.78 ± 3.31(89.96–95.59)	87.56 ± 4.56 (84.74–90.37)Ψ
15 min post	91.67 ± 4.53(86.22–97.12)	87.33 ± 9.92(81.88–92.78)
30 min post	90.11 ± 5.09(85.64–94.58)	82.89 ± 7.36(78.42–87.36 Ψ
	ELP (ul/l)	
	FAST	FED
Rest	107.56 ± 26.74(74.99–140.12)	129.22 ± 59.44(96.66–161.79)
15 min exercise	106.22 ± 22.45(80.60–131.85)¥	126.00 ± 46.11(100.37–151.63)
30 min exercise	106.11 ± 25.65(83.85–128.37)	119.89 ± 36.42(97.63–142.15)
Immediately post	98.44 ± 17.46(79.13–119.87)	118.33 ± 33.05(99.08–137.58)
15 min post	98.00 ± 17.46(76.13–119.87)	114.44 ± 40.13(92.58–136.31)
30 min post	92.33 ± 19.87(66.00–118.67)	116.67 ± 48.82(90.33–143.01)
	FFA (mmol/l)	
	FAST	FED
Rest	0.33 ± 0.16(0.21–0.46)	0.30 ± 0.20(0.18–0.43)
15 min exercise	0.28 ± 0.07€(0.24–0.32)€	0.14 ± 0.04(0.10–0.18)€ Ψ
30 min exercise	0.27 ± 0.05(0.24–0.30)€	0.14 ± 0.03(0.11–0.17)Ψ
Immediately post	0.43 ± 0.19(0.33–0.53)	0.17 ± 0.06(0.08–0.27)Ψ
15 min post	0.41 ± 0.08(0.36–0.46)	0.17 ± 0.05(0.12–0.22)Ψ
30 min post	0.37 ± 0.06(0.32–0.42)	0.16 ± 0.07(0.11–0.21)Ψ

FAST–Exercise performed in fasted condition; FED–Exercise performed after a breakfast.

* p<0.01 in relation to the other times

¥ p = 0.04 in relation to 15 min post-exercise

€ p<0.05 in relation with 15 min post-exercise

Ψ p<0.001 between FAST an FED

No moment nor exercise effects and no interaction moment x exercise were observed in ELP values.

In FAST it was observed a significant, (p = 0.04, CI95% = 0.83–26.95, d = 0.51), high value of ELP in the time 15 min exercise in relation to the moment 30 min post-exercise.

In relation to the FFA, a time effect (F_(5,80)_ = 5.166; p<0,001; η_p_^2^ = 0.244), an interaction time x condition effect (F_(5,80)_ = 3.966; p = 0.02; η_p_^2^ = 0.199) and an exercise effect (F_(1,16)_ = 29.646; p<0,001; η_p_^2^ = 0.699) were observed. In all the times, the FFA values were significantly (p<0.001, CI95% = 0.083–0.40, d = 1.85–3.60) higher in FAST compared with FED, with an exception at rest (see Table 5).

In FAST, FFA at 15 min exercise and at 30 min of exercise presented lower values compared with that at 15 min post-exercise (p = 0.01, CI95% = -0.24–-0.03, d = 1.73 and p = 0.03, CI95% = -0.26–-0.02, d = 2.10, respectively). In FED, FFA at 15 min exercise presented lower values compared with 15 min post-exercise (p = 0.02, CI95% = -0.06–-0.05, d = 0.66).

## Discussion

Absolute and relative VO_2_, HR, RER and the VO_2_ rec did not differ between conditions of exercise (FAST vs. FED). It has been previously identified that the exercises performed at the same intensity, volume and on the same device result in the same ventilatory and hemodynamic (HR) responses during [19] and after exercise [20]. However, in the present study, under FED conditions, the VO_2_ rec values remained higher for a longer period of time when compared with FAST (20 min post versus 5 min post, respectively). A possible explanation for this can be found in the thermogenic food effect, responsible for promoting higher values of VO_2_ in FED [21–23].

Regarding substrate utilization, during the 45-min walking in a treadmill, no difference in the substrate utilization between walking in fasting or fed was detected. Indeed, a predominance of carbohydrate utilization was observed in both conditions. Based on the your data, performing a continuous aerobic exercise in fasted condition does not appear to provide advantages compared to the same exercise performed under FED conditions. We do not know if the participants in the current study did exercised at their own fatmax intensity. Indeed, the participants of the present study were physically active and not endurance-trained individuals. Therefore, the intensity that corresponds to their maximal fat oxidation rate may be lower than some references in the literature [7,8]. However, since they exercised at the same intensity in both exercise condition, the above conclusions are safe for this subject cohort.

Testosterone levels did not differ between moments and conditions of exercise as they are directly connected to exercise intensity and volume [24–26]. The C values observed in the present study corroborate with those in previous studies, where prior food ingestion influenced a C level decrease [14,27–29].

Cortisol is a catabolic hormone that may increase the breakdown of muscular protein and its use as an energy substrate [30]. Over time, higher C levels could favour muscle mass loss, with a possible negative impact on weight loss, since muscle mass has an important influence on total daily energy expenditure [31]. The reduction of C levels in FED and its maintenance in FAST, may indicate that exercise with feeding is a better strategy to oxidize fat while, simultaneously, preserving muscle mass. Indeed, the higher T/C ratio in FED, when compared to FAST condition at 15-min post-exercise, may indicate a positive anabolic balance [32]. However, the present study can not anticipate how long these differences would last and whether the magnitude of elevation and interaction with time would result in any negative outcomes.

GLU levels remained stable during all periods of time measured in FAST [14,33]. This finding may be related to the plasma glucagon increase, that will stimulate hepatic glycogenolysis in order to maintain GLU within muscle cells, a necessity for exercise [34]. GLU levels were significantly (p<0.03) higher in FAST compared with FED at any moment that was analysed, with exception of rest and 15-min post-exercise. Also in FED, at 15-min during exercise the GLU values were significantly (p<0.05) lower compared with every other time. These results corroborate those found within the literature [34,35,36] and can be explained by the insulin action in response to the carbohydrates content in the previous meal (a shake with 32g of dextrose), promoting a reactive hypoglycemia [37].

A similar behavior of ELP was observed in both sessions (FAST and FED) of the present study. However, under FAST condition, at 15-min of exercise, ELP was significantly (p = 0.04) higher compared with that at 30 min post-exercise. The FFA blood concentrations at 15 and 30-min post-exercise in FAST and at 15-min post-exercise in FED were significantly (p<0.05) lower compared with that at 15 min post-exercise. Significantly higher FFA blood concentrations, (p<0.001) in FAST as compared to FED, were also found at 30-min of exercise and at 30-min post-exercise. These results corroborate those by Horowitz et al. [38], during cycling at 68% VO_2_ with a peak at 120 minutes, when comparing FFA blood concentrations between fasting and feeding conditions.

With a careful examination of the present study data, one might suggest that performing a low intensity aerobic exercise under FAST condition can promote a higher utilization of FFA [39]. Moreover, exercising muscles use FFA as a source of energy [40]. However, when the availability of FFA in blood exceeds the muscles´ ability to capture and oxidize this macronutrient, this surplus turns-over to the adipose tissue and is stored as triglyceride [5]. Even so, in the current study, no differences were found between FAST and FED in the ELP and fat used.

## Conclusion

In terms of fat oxidation, we conclude that performing a low intensity aerobic exercise in a fasting [FAST] condition does not seem to offer an advantage, as compared with performing the same exercise under a feeding [FED] condition. Moreover, based on Cortisol (C) levels, we also concluded that if a primary goal is to burn fat while, simultaneously, maintaining muscle mass, performing a low intensity aerobic exercise under a fasting condition might not be the best choice. These conclusions can only be extrapolated to a population with identical characteristics as that tested in the present study.

## Supporting information

S1 File Excel file presents all values of the dependent variables (VO2, energy substrates, hormones and enzymes) of each subject in each exercise condition as well as anthropometric measures. (XLSX)
